# The Story of the Insane from Year to Year

**Published:** 1900-03-17

**Authors:** 


### THE STORY OF THE INSANE FROM YEAR TO YEAR.

TWELFTH SERIES.

### THE GLAMORGAN COUNTY ASYLUM AT BRIDGEND.

ON January 1st, 1898, this asylum contained 1,484 patients, and by the end of December the numbers had risen to 1,575. There were 342 first admissions, 53 readmissions, 111 recoveries, 31 discharged relieved, and 151 deaths. The recoveries stand at 30·1 per cent. of the admissions, and the deaths at 8·9 per cent. of the average number resident. Of the total number of deaths 39 were caused by cerebral and spinal diseases, 26 by consumption, and 18 by influenza. Of the year's admissions 96 owed their insanity to moral causes, including domestic trouble, mental anxiety, and fright; 73 to intemperance in drink, and 129 to hereditary influence. During the year both temporary and permanent buildings were opened; but as the numbers increase by 100 a year, and as the Cardiff patients are to remain for five years while their own asylum is being built, more temporary wards are to be put up. Dr. Pringle expected a great decrease in the admissions subsequent on and consequent to the strike among the colliers; but although he was disappointed in this expectation he brings out the curious fact that while in 1897 there were 100 men admitted whose insanity was ascribed to drink, in 1898 there were only 56 from the same cause. And he further points out that the hereditary cases increased from 61 to 71. During the last few years the question of tuberculosis has sorely exercised the minds of our medical superintendents. Dr. Pringle thinks that in a considerable proportion of the cases in asylums the disease has originated in the asylum, and that the usual precautions should be insisted on, more especially as regards the separation of the infected from the healthy, the abundance of fresh air, and the quality of the milk given to the patients. The committee say that "Dr. Pringle continues to perform the arduous duties devolving on him with his usual fidelity, unflagging energy, and conspicuous ability." The average weekly cost per head is 8s. 5½d.

